# Liver Safety Assessment of an Indonesian Hexavalent Vaccine Candidate Through Histopathology and ALT/AST Evaluation in Rats and Rabbits

**DOI:** 10.3390/vaccines14010094

**Published:** 2026-01-19

**Authors:** Elisa D. Pratiwi, Tiza W. Mawaddah, Arif R. Sadjuri, Dimas T. Nugroho, Arip Hidayat, Astria N. Nidom, Zakiyyan I. Ayyuba, Eka S. Wahyuningsih, Kuncoro P. Santoso, Hani Plumeriastuti, Soeharsono, Setyarina Indrasari, Reviany V. Nidom, Acep R. Wijayadikusumah, Chairul A. Nidom

**Affiliations:** 1 Faculty of Veterinary Medicine, Universitas Airlangga, Surabaya 60115, Indonesia; 2 Professor Nidom Foundation, Surabaya 60236, Indonesia; 3 PT Bio Farma, Bandung 40161, Indonesia; 4 Faculty of Medicine, Universitas Airlangga, Surabaya 60132, Indonesia; 5 Global Biosains Teknologi, Malang 65145, Indonesia; 6 Riset AIRC Indonesia, Surabaya 60236, Indonesia

**Keywords:** hexavalent vaccine, toxicity test, alanine aminotransferase, aspartate aminotransferase, liver histopathology, rats, rabbits

## Abstract

Background: Administering several separate childhood vaccines can reduce adherence to immunization schedules due to missed appointments and the burden of repeated injections. A hexavalent formulation targeting diphtheria, tetanus, pertussis, hepatitis B, *Haemophilus influenzae* type B, and poliovirus offers a practical approach to improve compliance and streamline immunization. Methods: Toxicity testing was performed in Wistar rats and New Zealand White rabbits (60 rats and 30 rabbits). Animals were distributed into three groups: hexavalent vaccine + low-dose sIPV, hexavalent vaccine + high-dose sIPV, and control. Each animal received a 0.5 mL intramuscular injection at weeks 0, 4, 8, and 12. Clinical observations were conducted throughout the study. Serum samples were collected one day before each injection and at the endpoint, while liver tissue was collected at the endpoint. ALT and AST concentrations were analyzed using an automated analyzer, and hepatic morphology was evaluated microscopically. Results: No abnormal clinical signs related to vaccination were observed. ALT concentrations showed no significant differences (*p* > 0.05). AST differences (*p* < 0.05) were detected between the high-dose group and the control on day 27 in female rabbits and on day 83 in female rats; however, all values remained within normal physiological limits. Histopathological examination revealed no irreversible hepatic lesions, including hydropic degeneration, portal inflammation, focal necrosis, or connective tissue proliferation, and no significant differences were noted (*p* > 0.05). Conclusions: Repeated administration of the hexavalent vaccine candidate at low and high doses produced no toxicological effects in animal models, supporting its safety for further clinical development.

## 1. Introduction

The wide variety of vaccines that must be administered during childhood often leads to non-compliance with recommended immunization schedules, which may arise from caregiver negligence or the psychological burden associated with repeated injections. Combination vaccines have therefore become an important strategy to improve adherence and streamline the immunization process by delivering multiple antigens in a single dose. In addition, these formulations are expected to reduce healthcare costs by minimizing expenditures related to administration and logistical needs [1]. Indonesia has not yet released official national coverage data for tetanus, diphtheria, pertussis, hepatitis B, *Haemophilus influenzae* type b (Hib), and polio vaccines for 2025. Nevertheless, WHO-UNICEF (WUENIC) estimates for 2023–2024 indicate that routine immunization coverage remains relatively strong. DTP3 coverage is approximately 81%, while HepB3 ranges from 62% to 84%, depending on survey methodology. Hib3 coverage, delivered through the pentavalent DTP-HB-Hib vaccine, is estimated at around 66%, and Pol3 coverage ranges from 81% to 86% [2,3].

Despite these encouraging national figures, timely vaccination remains a concern. One study reported that 67% of infants received at least one dose outside the recommended schedule, with delays influenced by several factors. Infants born in private healthcare facilities had nearly twice the likelihood of experiencing delayed immunization, and those residing in rural areas were also at a higher risk of late vaccination [4]. These gaps in timely uptake highlight the need for strategies that can simplify vaccine delivery. In this context, the development of hexavalent vaccines represents a promising advancement in immunization technology. Before distribution, each vaccine candidate must undergo a series of rigorous quality control steps, including pre-clinical and clinical evaluation, regulatory review, and the release of the first approved batch, before it can be made available for use [5].

Several factors can influence the toxicity, efficacy, and effectiveness of vaccines, including vaccine components (antigens, excipients, and adjuvants), interactions among these components, the route and frequency of administration (boosters), and the dosage used [6]. Preclinical evaluation of vaccines using animal models is expected to demonstrate immune responses comparable to those observed in humans following vaccination. In general, a single relevant animal species may be sufficient for toxicity testing. However, in this study, two animal models, rodent, and non-rodent were employed to anticipate potential species-specific pharmacodynamic differences. This approach is consistent with the World Health Organization (WHO) guidelines (TRS No. 927, Annex 1, and No. 987, Annex 2), which recommend the use of more than one animal species when the mechanism of action of the vaccine is not yet fully understood or when species-specific responses to the vaccine product may occur [7,8].

Understanding interspecies variation in molecular targets is essential for interpreting toxicological outcomes and predicting potential human risk. Molecules that modulate the immune system in one species generally exert comparable biological effects in others, although qualitative and quantitative differences may occur [9]. Rats are among the most widely used models in biomedical and toxicological studies due to their well-characterized physiology and relevance to human health [10,11], while New Zealand White rabbits are frequently employed because of their physiological similarities to humans. Evaluation of physiological, hematological, and biochemical parameters is fundamental for assessing homeostatic disturbances and identifying early pathological changes across organ systems [12,13,14]. The liver, the body’s largest internal organ, plays central roles in metabolism, storage, synthesis, and detoxification, making it particularly vulnerable to xenobiotic-induced injury. Hepatocellular damage may arise from direct toxicant exposure or immune-mediated mechanisms involving innate or adaptive responses [15], typically reflected by increases in serum alanine aminotransferase (ALT) and aspartate aminotransferase (AST).

With recent advancements in multivalent vaccine technologies, there is an increasing need for comprehensive preclinical safety evaluations. Although ALT and AST serve as sensitive biomarkers of hepatic functional alterations, biochemical measurements alone may not detect subtle structural abnormalities. Therefore, histopathological assessment remains essential for identifying microscopic hepatic lesions that may not be captured through routine serum enzyme analyses. However, integrated evaluations combining biochemical and histopathological endpoints for hexavalent vaccine candidates are still limited, and no published work has assessed the hepatic safety of a hexavalent vaccine with this antigenic composition using complementary rodent and non-rodent models. To address this gap, the present study evaluates the hepatic safety profile of a hexavalent vaccine candidate in Wistar rats (*Rattus norvegicus*) and New Zealand White rabbits (*Oryctolagus cuniculus*) through a multi-parameter assessment comprising clinical observations, ALT and AST activities, and detailed liver histopathology.

## 2. Materials and Methods

### 2.1. Vaccine

The hexavalent vaccine (DTwP-HepB-Hib-IPV) used in this study was manufactured by PT Bio Farma (Persero), Bandung, Indonesia. Dose levels were selected based on preliminary safety data and standard dose-multiplying principles used in vaccine toxicity testing. The vaccine formulation contained an aluminium (Al^3+^) adjuvant at a concentration of 0.33 mg per dose. The target antigen composition per dose was as follows: diphtheria antigen (D) ≥ 60 IU, tetanus antigen (T) ≥ 40 IU, whole-cell pertussis antigen (wP) ≥ 4 IU, hepatitis B antigen ≥ 10 µg, and *Haemophilus influenzae* type b (Hib) antigen ≥ 10 µg. The concentrations of these antigens were identical in both the low-dose and high-dose hexavalent vaccine formulations. The only difference between the two formulations was the content of the inactivated poliovirus (sIPV) component. The high-dose formulation contained sIPV with a potency of 15-45-45 DU per dose for poliovirus types 1, 2, and 3, respectively, whereas the low-dose formulation contained two-thirds (2/3) of the sIPV dose used in the high-dose formulation.

The formulation process was initiated by mixing aluminum phosphate adjuvant with hepatitis B, tetanus, and diphtheria antigens under controlled conditions, followed by continuous stirring until a homogeneous mixture was obtained. Subsequently, whole-cell pertussis, Hib, and sIPV antigens (types 1, 2, and 3) were added sequentially. Water for Injection (WFI) was then added to achieve the target formulation volume. Final mixing and pH adjustment were performed at the last stage of formulation to ensure that the pH of the final vaccine product was within the range of 6.0–7.5.

### 2.2. Test Animals

This study was conducted in accordance with the World Health Organization (WHO) guidelines (TRS No. 927, Annex 1, and TRS No. 987, Annex 2). A total of 60 Wistar rats (10 males and 10 females per group) and 30 New Zealand White rabbits (5 males and 5 females per group) were used. Animals were randomly allocated to control and treatment groups following a two-week acclimatization period to ensure balanced distribution among groups. The New Zealand White rabbits were 3–4 months old, with body weights ranging from 1.700 to 2.500 g, while the Wistar rats were 6–8 weeks old and weighed between 175 and 250 g. Animals were included in the study if they were clinically healthy, within the predefined age and body weight ranges, and showed no signs of disease, particularly those related to the experimental treatment. The animals were housed in an accredited animal laboratory facility (ABSL-3) under controlled environmental conditions, with a temperature of 24 °C and relative humidity of 60%. Animals had ad libitum access to food and water. All animal procedures in this study were conducted in compliance with national laws and institutional guidelines for the care and use of laboratory animals. The study protocol was reviewed and approved by the Animal Care and Use Committee (Komite Etik Penggunaan Hewan Percobaan) Institut Teknologi Bandung (Approval No. KEP/I/2024/VIII/H1507241K/TSVT).

### 2.3. Experimental Design

Group 1 (P1) received the hexavalent vaccine containing sIPV at a low dose, Group 2 (P2) received the hexavalent vaccine containing sIPV at a high dose, and Group 3 (P3) served as the control and was administered phosphate-buffered saline (PBS). The study was conducted as an 87-day repeated-dose subchronic toxicity assessment. Vaccination was initiated on day 0, followed by booster doses at weeks 4, 8, and 12, reflecting the human immunization schedule. All injections were administered intramuscularly into the thigh muscle. Rabbits received a single injection (0.5 mL), whereas rats received bilateral injections (0.25 mL) per site.

Blood samples were collected one day prior to each vaccination (weeks 0, 4, 8, and 12) and at the study endpoint. At the endpoint, all animals were humanely euthanized, and serum and liver tissue samples were collected for histopathological evaluation. Euthanasia was performed by administering an overdose of anesthetic agents. Rabbits received intramuscular ketamine-xylazine (35 mg/kg-5 mg/kg), while rats received intraperitoneal ketamine-xylazine (75 mg/kg-10 mg/kg) [16].

### 2.4. Clinical Observations Procedures

Physical parameters monitored included general condition, locomotion, mucosal color, lacrimation, salivation, respiratory rate, heart rate, tremors, convulsions, vomiting, fecal abnormalities, and urination. In this exploratory toxicology study, clinical signs, body weight, hepatic enzyme activities (ALT and AST), and liver histopathology were systematically assessed.

### 2.5. Biochemical Analysis

Biochemical parameters included alanine aminotransferase (ALT) and aspartate aminotransferase (AST) levels, which were analyzed to assess potential liver injury and liver function following vaccine administration. Enzyme activities were determined using commercially available assay kits (Glory Diagnostic, Barcelona, Spain) according to the manufacturer’s instructions, and absorbance was measured using a Tecan Infinite 200 Pro microplate reader (Tecan Group Ltd., Männedorf, Switzerland).

### 2.6. Histopathological Analysis

Histopathological preparations were obtained from the liver tissues of rabbits and rats. These tissues were stained using Hematoxylin and Eosin (H&E) staining. In brief, small fragments of fresh tissue were fixed in 10% buffered formalin, then gradually dehydrated, embedded in paraffin, sectioned at 5 µm thickness, deparaffinized with p-xylene, rehydrated through graded ethanol, rinsed in water, and finally stained with Hematoxylin and Eosin (H&E) [17]. Histopathological observations were performed using a Nikon^®^ Eclipse C1 (Nikon Corporation, Tokyo, Japan).

Tissue damage classification was based on a modified scoring system developed by Knodell et al. (1981) [18], with specific adjustments. Intralobular hepatic degeneration and focal necrosis were graded as follows: none (score 0), mild (<1/3 of the lobule; score 1), moderate (1/3–2/3 of the lobule; score 3), and severe (>2/3 of the lobule; score 4). Portal inflammation was graded as: no inflammation (score 0), mild (inflammatory cells occupying <1/3 of the portal area; score 1), moderate (1/3–2/3 of the portal area; score 3), and severe (>2/3 of the portal area; score 4).

### 2.7. Data Analysis

Data analysis was expressed as means with standard deviations (SD). Data obtained were statistically analyzed using SPSS software (version 27). Analysis of Variance (ANOVA) was performed to determine significant differences among treatment groups, followed by Duncan’s multiple range test for post hoc comparisons when applicable (*p <* 0.05). The results of hepatic degeneration, focal necrosis and portal inflammatory cell infiltration scoring were analyzed using ranking assessment. Quantitative data were analyzed using non-parametric statistical tests (Kruskal–Wallis test), while qualitative data were analyzed descriptively and further evaluated using the Mann–Whitney test to identify differences between groups.

## 3. Results

### 3.1. Body Weight

**Figure 1:**
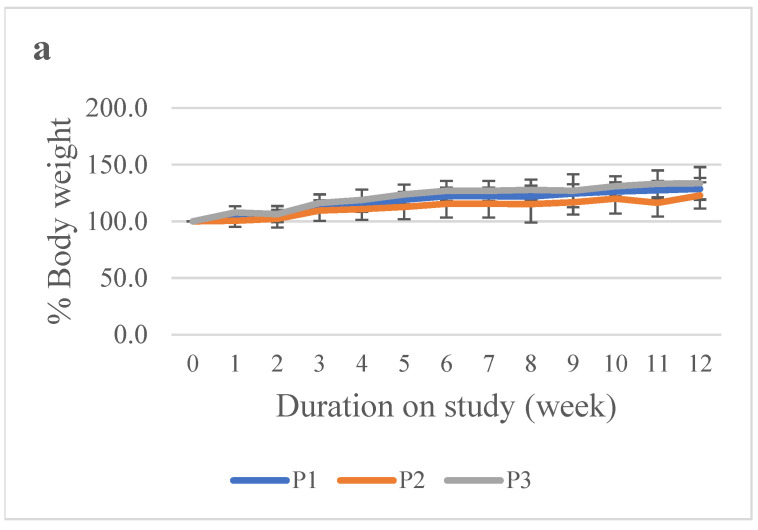

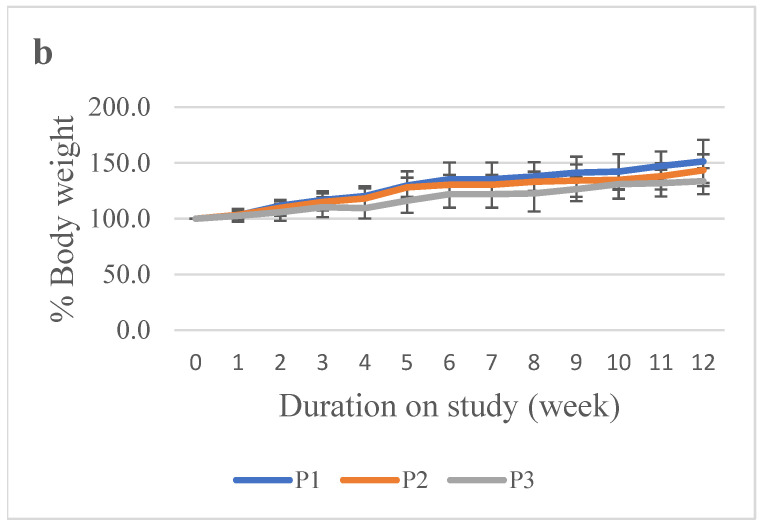
Percentage body weight of rabbits in the repeated-dose toxicity study of the hexavalent vaccine candidate. (**a**) Male rabbits; (**b**) Female rabbits. Animals were injected at weeks 0, 4, 8, and 12 and monitored weekly throughout the study period to observe any changes in body weight.

The percentage body weight of both male and female rabbits in all treatment groups (P1, P2, and P3) showed a consistent upward pattern throughout the observation period (weeks 0–12). At the beginning of the study, body weights were approximately 100% of baseline and gradually increased toward the end of the study. All three groups exhibited a similar trend, with no meaningful decrease in body weight at any time point (Figure 1).

**Figure 2:**
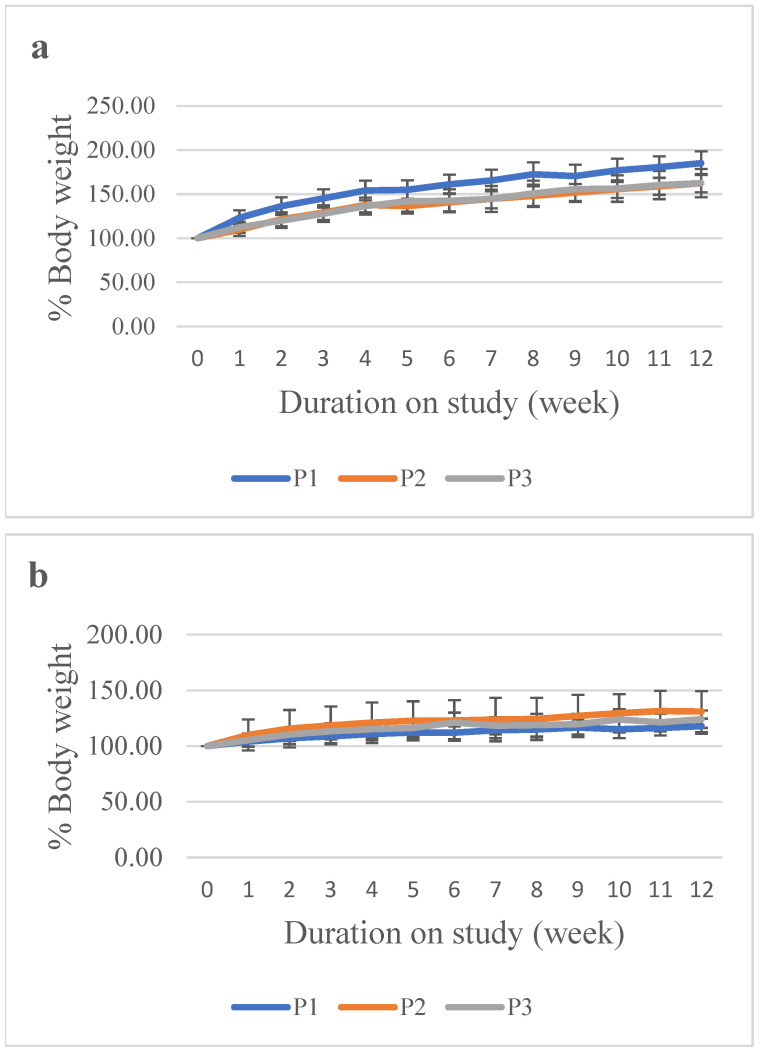
Percentage body weight of rats in the repeated-dose toxicity study of the hexavalent vaccine candidate. (**a**) Male rats; (**b**) Female rats. Animals were injected at weeks 0, 4, 8, and 12 and monitored weekly throughout the study period to observe any changes in body weight.

The percentage body weight of male and female rats in the treatment groups (P1 and P2) demonstrated a consistent upward pattern throughout the observation period. Both groups exhibited comparable trends, with no meaningful reductions in body weight at any measurement point. Rats in the high-dose group showed a higher mean body weight compared to the control group. However, the increase observed in P2 remained within a narrow range and did not exceed twice the normal values, indicating that the change was still within normal physiological limits (Figure 2).

### 3.2. Clinical Observations

No deaths of the rabbits and rats were found during the study. None of the animals showed clinical symptoms that indicated illness. All physical parameters, including general condition, locomotion, mucosal color, lacrimation, salivation, respiratory rate, heart rate, tremors, convulsions, vomiting, fecal characteristics, and urination, remained within normal limits.

#### 3.2.1. Alanine Aminotransferase (ALT)

**Figure 3:**
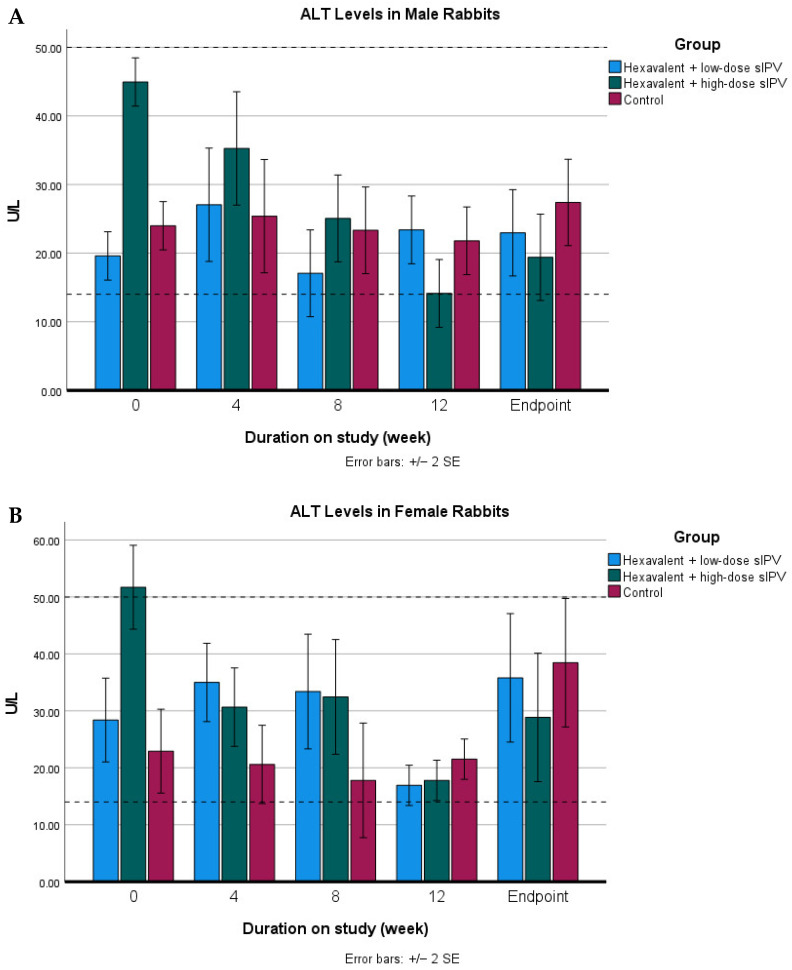

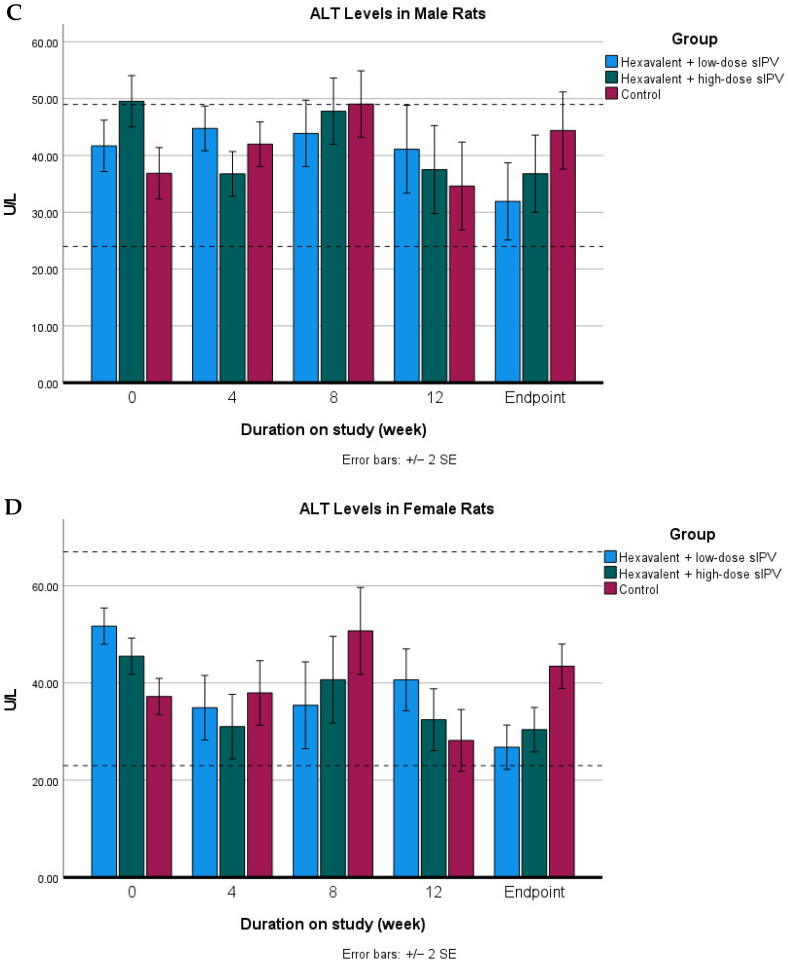
ALT values in rabbits and rats in the repeated-dose toxicity study of the hexavalent vaccine candidate. Animals were injected at weeks 0, 4, 8, and 12 and were monitored one day prior to each injection for blood sample collection and at the study endpoint. The figure presents ALT values for male rabbits (**A**), female rabbits (**B**), male rats (**C**), and female rats (**D**). Dashed horizontal lines indicate the lower and upper reference limits for ALT.

The statistical analysis revealed no significant interaction (*p* > 0.05) in ALT concentrations with respect to the experimental animal model, sex, treatment, or observation period (week), as presented in Figure 3.

#### 3.2.2. Aspartate Aminotransferase (AST)

**Figure 4:**
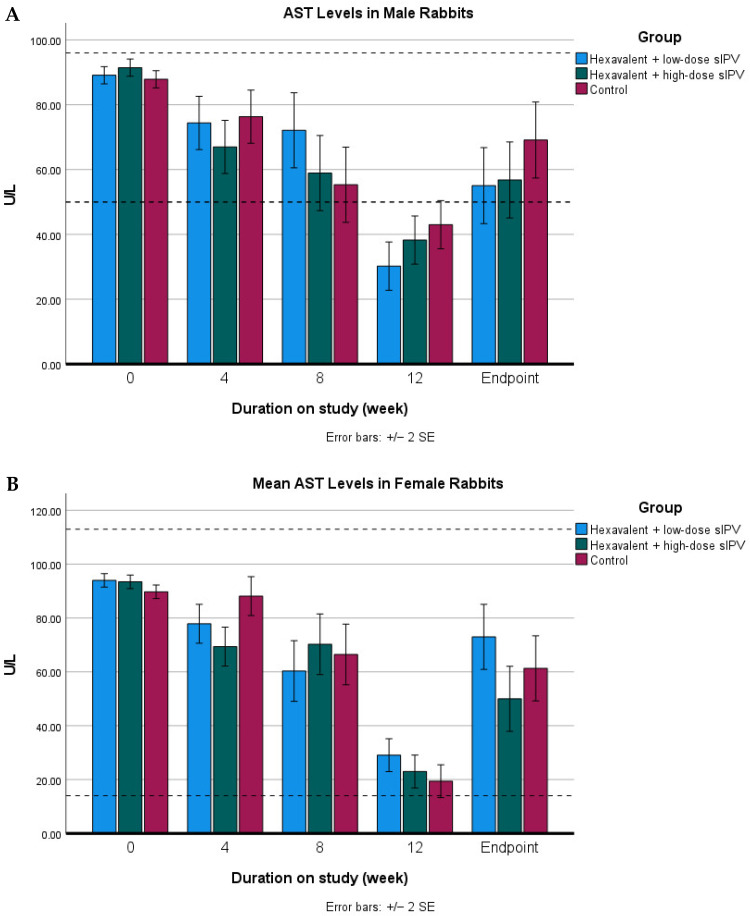

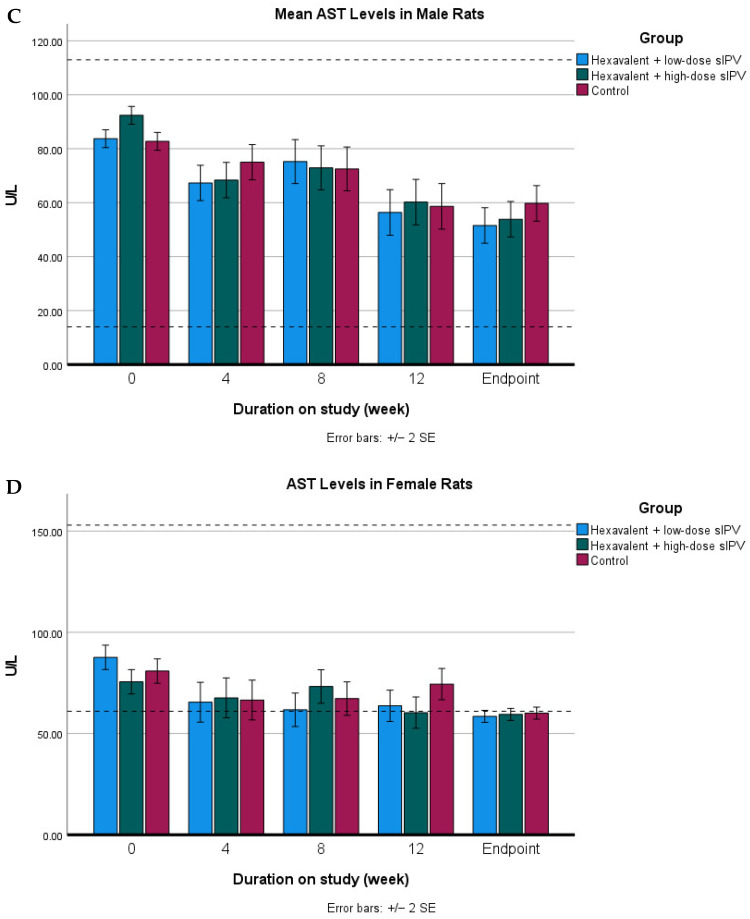
AST values in rabbits and rats in the repeated-dose toxicity study of the hexavalent vaccine candidate. Animals were injected at weeks 0, 4, 8, and 12 and were monitored one day prior to each injection for blood sample collection and at the study endpoint. The figure presents AST values for male rabbits (**A**), female rabbits (**B**), male rats (**C**), and female rats (**D**). Dashed horizontal lines indicate the lower and upper reference limits for AST.

The statistical analysis revealed a significant interaction (*p* < 0.05) among the experimental animal model, sex, treatment, and observation period (week) for AST concentrations. Post hoc analysis using Duncan’s multiple range test indicated significant differences (*p* < 0.05) between P2, which received the high-dose hexavalent vaccine containing sIPV, and P3, the control group, specifically at week 4 in female rabbits and at week 12 in female rats. However, the magnitude of the AST changes did not exceed twice the normal reference values, indicating that these variations remained within normal physiological limits, as shown in Figure 4.

### 3.3. Effect of Hexavalent Vaccine on Clinical Pathology and Histopathology Changes

Macroscopic examination of the liver revealed no visible changes or lesions in either rabbits or rats across all treatment groups.

### 3.4. Intralobular Hepatic Degeneration and Focal Necrosis

**Figure 5:**
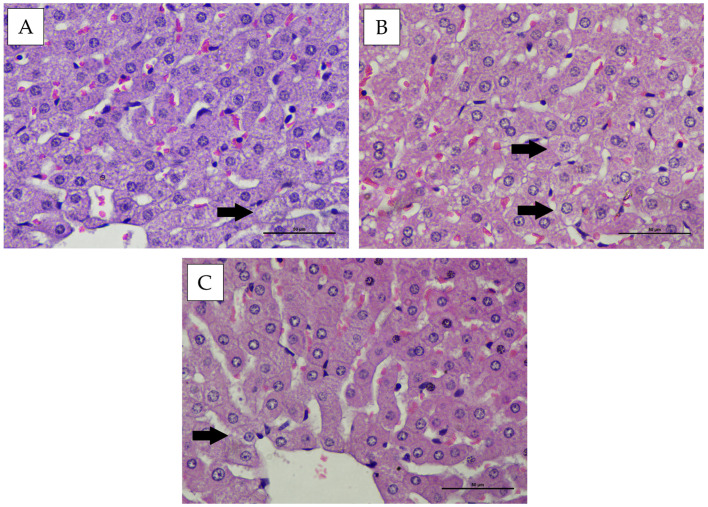
Histopathological appearance of the hepatic tissue in New Zealand White rabbits following treatment, stained with Hematoxylin and Eosin (H&E) (400× magnification). (**A**) group P1, (**B**) group P2, (**C**) group P3. Arrows indicate hepatocytes exhibiting hydropic degeneration.

**Figure 6:**
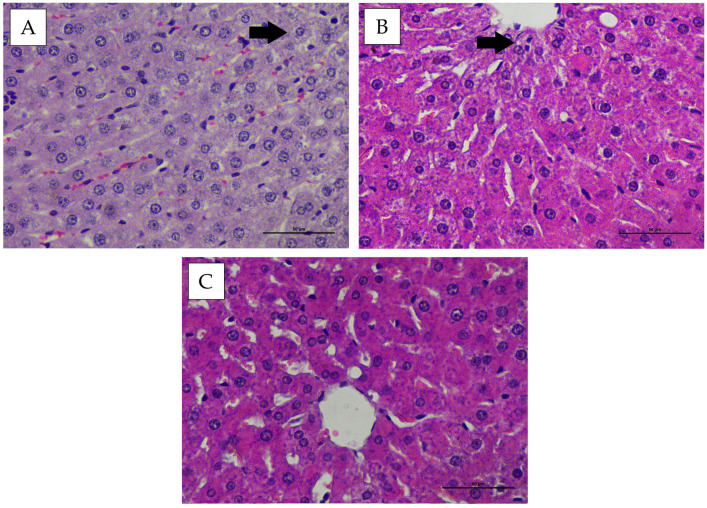
Representative histopathological appearance of liver tissue from Wistar rats following treatment, stained with Hematoxylin and Eosin (H&E) (400× magnification). (**A**) group P1, (**B**) group P2, (**C**) group P3. Arrows indicate hepatocytes exhibiting hydropic degeneration.

Histopathological evaluation of the intralobular hepatic region in New Zealand White rabbits and Wistar rats showed mild hepatocellular degeneration in several animals. The hepatocytes appeared swollen with cloudy cytoplasm, while the nuclei remained morphologically normal in all treatment and control groups following administration of the hexavalent vaccine candidate (Figure 5 and Figure 6). The microscopic examination of the liver also revealed no evidence of necrosis in any observed field (Figure 5 and Figure 6). Statistical analysis of intralobular hepatic degeneration and focal necrosis using the Kruskal–Wallis test demonstrated no significant differences (*p* > 0.05) among the experimental groups (Table 1 and Table 2).

### 3.5. Portal Hepatic Inflammation

**Figure 7:**
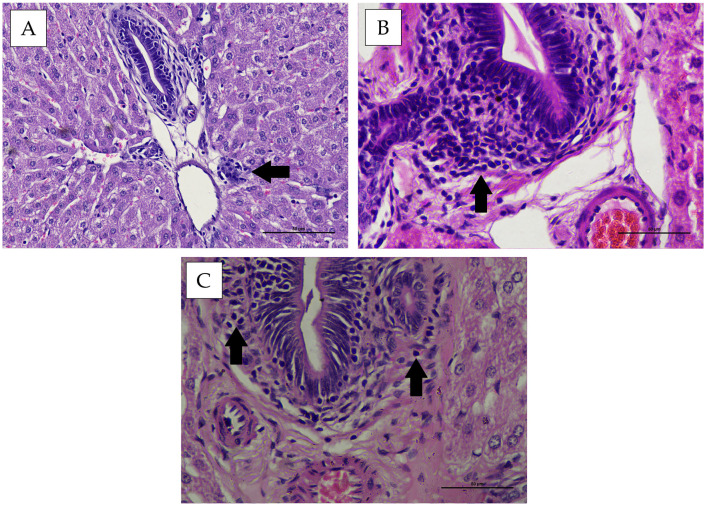
Representative histopathological appearance of liver tissue from New Zealand White rabbits following treatment, stained with Hematoxylin and Eosin (H&E) (400× magnification). (**A**) group P1, (**B**) group P2, (**C**) group P3. Arrows indicate hepatocytes exhibiting cell infiltration.

**Figure 8:**
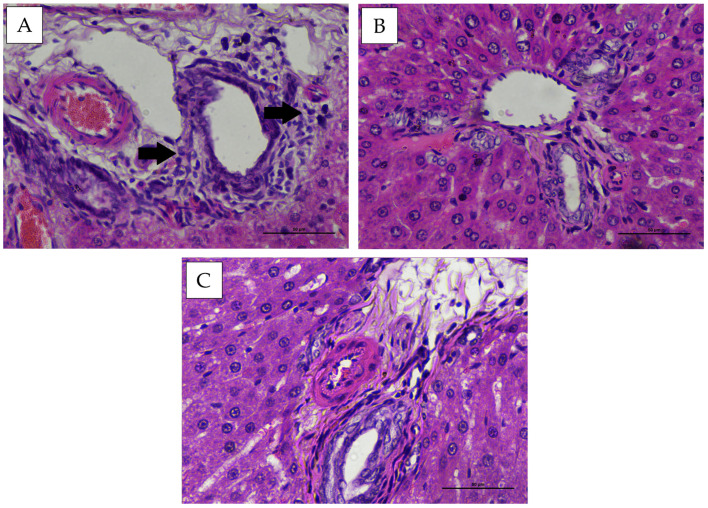
Representative histopathological appearance of liver tissue from Wistar rats following treatment, stained with Hematoxylin and Eosin (H&E) (400× magnification). (**A**) group P1, (**B**) group P2, (**C**) group P3. Arrows indicate hepatocytes exhibiting cell infiltration.

Histopathological observation of the hepatic portal area in New Zealand White rabbits and Wistar rats showed mild infiltration of inflammatory cells in some animals. No fibrotic tissue formation was observed microscopically (Figure 7 and Figure 8). Statistical evaluation of portal hepatic inflammation revealed no significant differences (*p* > 0.05) between animal species, sexes, or treatment groups (Table 3).

## 4. Discussion

Vaccination coverage must exceed 95% of the total population to be effective in preventing outbreaks of infectious diseases [19]. Although effective vaccines are available, vaccine hesitancy defined as the refusal or delay in accepting vaccination, remains a major concern [20]. Immunization with minimal adverse effects following vaccination remains a major public concern [21]. Therefore, preclinical toxicity testing of vaccines is conducted to identify any potential toxic effects of the vaccine candidate, thereby allowing its safety to be assessed prior to progression to the next stage, namely clinical trials. The vaccine candidate examined in this study is a hexavalent vaccine containing the antigens DTwP-HepB-Hib-sIPV, which provides protection against Diphtheria, Tetanus, Pertussis, Hepatitis B, *Haemophilus influenzae* type B (Hib), and Poliovirus types 1, 2, and 3.

We evaluated the safety of the hexavalent vaccine candidate through an 87-day repeated subchronic toxicity study by assessing clinical observations, body weight, biochemical parameters of liver function, and histopathological findings of the liver. Based on clinical observations, no clinical signs or mortality were detected throughout the study. The body weights of both rabbits and rats also increased from the pre-vaccination period to the end of the study. The increase in body weight observed in the treatment groups aligns with findings from previous vaccine toxicity studies. Experimental data generally indicate that vaccination does not lead to weight loss in laboratory animals. In several reports, rodents immunized with various vaccines including oral polio, diphtheria-pertussis-tetanus, bacillus Calmette-Guérin, measles, yellow fever, and hepatitis B, continued to gain weight following immunization, suggesting that the vaccines did not adversely affect growth parameters [22,23].

Blood chemistry serves as an essential diagnostic indicator for assessing the physiological condition of experimental animals. Among the key parameters, the biochemical profile of hepatic function can be evaluated based on the activity of enzymes such as alanine aminotransferase (ALT) and aspartate aminotransferase (AST). Damage to hepatocyte cells leads to a significant increase in ALT and AST levels in the bloodstream. ALT is considered a more specific and reliable biomarker for assessing hepatocellular integrity compared with AST [24], as ALT is predominantly concentrated in liver tissue, particularly within hepatocyte mitochondria [25,26]. Increases in ALT are typically more pronounced in cases of hepatic injury.

In the present study, ALT levels in all experimental groups remained within the normal reference ranges after vaccination, with no statistically significant differences observed between groups (*p* > 0.05). This finding suggests that the administered vaccine was not associated with hepatocellular injury, as clinically relevant liver damage is typically associated with ALT elevations exceeding twice the normal reference values. Consistent with previous reports, rabbits with liver disorder may exhibit clinical signs ranging from mild lethargy to more serious illness. Reported reference ranges for ALT in New Zealand White rabbits vary across the literature, with values ranging from 14 to 18 U/L [27] to 15–50 [28], while normal ALT values in Wistar rats have been reported to range from 24 to 49 U/L in males and 23–67 U/L in females [29].

In contrast, AST parameters showed a significant difference compared with the control group in the high-dose group (P2) of female rabbits at week 4 and female rats at week 12. However, when analyzed based on reference ranges reported in previous studies the AST levels in New Zealand White rabbits ranged from 14 to 113 U/L [27]. Similarly, normal AST levels in rats range from 50 to 96 U/L in males and 61–153 U/L in females [29]. Therefore, although statistically significant, the observed AST alterations were not indicative of pathological liver injury. Moreover, no consistent increasing trend in AST activity was observed across other observation time points, suggesting that the changes were transient rather than progressive in nature. Such variations may reflect interspecies differences, sex-related physiological variability, or individual biological responses among experimental animals rather than treatment-related toxicity.

Aspartate aminotransferase (AST) is a transaminase enzyme found in various tissues, including the heart, liver, skeletal muscles, kidneys, brain, pancreas, and spleen. Within the liver, approximately 70% of AST is localized in the mitochondrial fraction of hepatocytes, while the remaining 30% is present in the cytoplasmic fraction [] [30]. Due to this broad tissue distribution, isolated alterations in AST activity do not necessarily reflect hepatocellular injury [25]. Under normal physiological conditions, serum AST concentrations remain relatively low because this enzyme is predominantly intracellular. In addition, histopathological evaluation of liver tissues was performed to confirm the absence of structural alterations indicative of hepatotoxicity [31].

Based on histopathological evaluation, inflammatory cell infiltration was observed within the hepatic portal area. Viriyavejakul et al. (2014) reported that portal inflammatory infiltrates predominantly consist of lymphocytes and plasma cells. Inflammation is a dynamic process, with its severity largely depending on the nature of the inducing agent and the host immune response [32]. It can trigger cellular alterations such as degeneration or necrosis. Both polymorphonuclear and mononuclear leukocytes can be activated by advanced glycation end products, oxidative stress, angiotensin II, and cytokines produced under hyperglycemic conditions [33]. Vaccine components may exert specific mechanisms that influence hepatic function, such as viral vectors or adjuvants acting directly on hepatocytes [34]. Immune activation against viral proteins may mediate immune reactions leading to hepatic inflammation [35].

Portal inflammation observed in this study ranged from mild to moderate, with some individuals showing no histological evidence of inflammation. This response is likely attributable to immune activation triggered by cytokine release. Such reactions typically become evident around day 14 post-antigen administration, marked by the presence of immune cells such as macrophages and lymphocytes, followed by the production of pro-inflammatory cytokines [36]. The histopathological features of the hepatic portal area also revealed physiological congestion, characterized by preserved vascular architecture without notable leakage, and the absence of significant inflammation or tissue edema. These observations are consistent with the description by Adil and Somanath [37] Physiological congestion represents a normal hepatic response to hemodynamic shifts, increased metabolic demand, or ongoing tissue recovery. This type of congestion is typically transient and does not result in permanent structural injury to the liver.

Histopathological examination revealed that hepatocellular degeneration was limited to mild swelling, with no evidence of hydropic degeneration or cell ballooning across all microscopic fields. Degeneration of the hepatic parenchyma is characterized by swelling and cytoplasmic cloudiness resulting from the formation of granules derived from protein accumulation. This condition is considered reversible, as it involves damage limited to cellular organelles such as mitochondria and the endoplasmic reticulum [38]. Moreover, no evidence of necrosis was observed in any microscopic field of the hepatic tissue. Although mild pathological changes, including degeneration and portal inflammation, were detected across all treatment groups, the Kruskal–Wallis test revealed no statistically significant differences between the dose groups and the control group. These findings indicate that both the hexavalent vaccine with SIPV low and high doses did not induce severe hepatic injury. In addition, no consistent alterations were observed in either species or sex. Therefore, these lesions are regarded as spontaneous or incidental findings and are not considered related to the administration of the hexavalent vaccine candidate.

## 5. Conclusions

The results of this study offer corroborative evidence supporting the hepatic safety profile of the hexavalent vaccine candidate, as reflected by liver function parameters and histopathological findings. While the data obtained from rabbits and rats cannot be directly extrapolated to humans, they provide a critical foundation for progressing the safety evaluation of the vaccine candidate toward clinical testing.

## Figures and Tables

**Table 1:** Mean rank values of intralobular hepatic degeneration in New Zealand White rabbits and Wistar rats following vaccination with the hexavalent vaccine candidate.

Group	Mean Rank
P1	46.97
P2	47.55
P3	41.98

**Table 2:** Mean rank values of focal necrosis in New Zealand White rabbits and Wistar rats following vaccination with the hexavalent vaccine candidate.

Group	Mean Rank
P1	45.50
P2	45.50
P3	45.50

**Table 3:** Mean rank values of portal hepatic inflammation in New Zealand White rabbits and Wistar rats following vaccination with the hexavalent vaccine candidate.

Group	Mean Rank
P1	33.75
P2	33.50
P3	24.25

## Data Availability

Data will be made available on request.

## References

[1] Özen M., Ünüvar E., Yıldırım A., Akman H., Mevlitoğlu S., Pehlivan T. (2024). A Worldwide Overview for Hexavalent Vaccines and A Glimpse into Turkiye’s Perspective. Hum. Vaccines Immunother..

[2] World Health Organization, United Nations Children’s Fund (2024). Notes on WHO–UNICEF Estimates of National Immunization Coverage (2024 Revision).

[3] World Health Organization (2023). Haemophilus Influenzae Type b (Hib) Infection.

[4] Oktaria V., Bines J.E., Murni I.K., Dinari R., Indraswari B.W., Alvianita A., Putri D.A., Danchin M. (2022). Timeliness of Routine Childhood Vaccinations in Indonesian Infants in the First Year of Life. Vaccine.

[5] Orsi A., Azzari C., Bozzola E., Chiamenti G., Chirico G., Esposito S., Francia F., Lopalco P., Prato R., Russo R. (2018). Hexavalent Vaccines: Characteristics of Available Products and Practical Considerations from a Panel of Italian Experts. J. Prev. Med. Hyg..

[6] Kocourkova A., Honegr J., Kuca K., Danova J. (2017). Vaccine Ingredients: Components That Influence Vaccine Efficacy. Mini Rev. Med. Chem..

[7] World Health Organization (2005). WHO Guidelines on Non-Clinical Evaluation of Vaccines, TRS No. 927. https://www.who.int/publications/m/item/nonclinical-evaluation-of-vaccines-annex-1-trs-no-927.

[8] World Health Organization (2014). Guidelines on the Non-Clinical Evaluation of Vaccine Adjuvants and Adjuvanted Vaccines, TRS No. 987. https://www.who.int/publications/m/item/nonclinical-evaluation-of-vaccine-adjuvants-and-adjuvanted-vaccines-annex-2-trs-no-987.

[9] Evans E.W., Casinghino S. (2018). Clinical Pathology as a Tool to Assess Immunotoxicity. Comprehensive Toxicology.

[10] Branco A.C.S.C., Diniz M.F.F.M., Almeida R.N., Santos H.B., Oliveira K.M., Ramalho J.A., Dantas J.G., Diniz M.F.F.M., Almeida R.N., Santos H.B. (2011). Biochemical and Hematological Parameters of Wistar Rats and Swiss Mice in the Professor Thomas George Animal Laboratory. Rev. Bras. Ciênc. Saúde.

[11] Otto G.P., Rathkolb B., A Oestereicher M., Lengger C.J., Moerth C., Micklich K., Fuchs H., Gailus-Durner V., Wolf E., Angelis M.H. (2016). Clinical Chemistry Reference Intervals for C57BL/6J, C57BL/6N, and C3HeB/FeJ Mice (*Mus musculus*). J. Am. Assoc. Lab. Anim. Sci..

[12] Almeida A.S., Faleiros A.C.G., Teixeira D.N.S., Cota U.A., Chica J.E.L. (2008). Reference Values for Blood-Based Biochemical Parameters in BALB/c and C57BL/6 Wild-Type Mice. J. Bras. Patol. Med. Lab..

[13] Restell T.I., Porfirio L.C., Souza A.S., Silva I.S. (2014). Hematology of Swiss Mice (*Mus musculus*) of Both Genders and Different Ages. Acta Cir. Bras..

[14] Silva-Santana G., Bax J.C., Fernandes D.C.S., Bacellar D.T.L., Hooper C., Dias A.A.S.O., Silva C.B., Souza A.M., Ramos S., Santos R.A. (2020). Clinical Hematological and Biochemical Parameters in Swiss, BALB/c, C57BL/6 and B6D2F1 *Mus musculus*. Anim. Models Exp. Med..

[15] Ye H., Nelson L.J., Gómez Del Moral M., Martínez-Naves E., Cubero F.J. (2018). Dissecting the Molecular Pathophysiology of Drug-Induced Liver Injury. World J. Gastroenterol..

[16] Flecknell P. (2015). Laboratory Animal Anaesthesia.

[17] Momtazi-Borojeni A.A., Banach M., Tabatabaei S.A., Sahebkar A. (2023). Preclinical Toxicity Assessment of a Peptide-Based Anti-PCSK9 Vaccine in Healthy Mice. Biomed. Pharmacother..

[18] Knodell R.G., Ishak K.G., Black W.C., Chen T.S., Craig R., Kaplowitz N., Kiernan T.W., Wollman J. (1981). Formulation and Application of a Numerical Scoring System for Assessing Histological Activity in Asymptomatic Chronic Active Hepatitis. Hepatology.

[19] Bechini A., Boccalini S., Ninci A., Zanobini P., Sartor G., Bonaccorsi G., Grazzini M., Bonanni P. (2019). Childhood Vaccination Coverage in Europe: Impact of Different Public Health Policies. Expert Rev. Vaccines.

[20] World Health Organization (2019). Ten Health Issues WHO Will Tackle This Year. https://www.who.int/news-room/spotlight/ten-threats-to-global-health-in-2019.

[21] Erener S. (2020). Diabetes, Infection Risk and COVID-19. Mol. Metab..

[22] Oli A.N., Agu R.U., Oli U.C., Nwoye C.U., Ejiofor O.S., Esimone C.O. (2015). Safety Evaluation in Mice of the Childhood Immunization Vaccines from Two South-Eastern States of Nigeria. Asian Pac. J. Trop. Biomed..

[23] Oli A.N., Oli U.C., Ejiofor O.S., Nwoye C.U., Esimone C.O. (2016). An Assessment, in Mice, of the Safety of the Childhood Immunization Vaccines Sourced from Three South-Eastern States of Nigeria. Trials Vaccinol..

[24] Fernandes D.P., Pimentel M.M., DOS Santos F.A., Praxedes É.A., DE Brito P.D., Lima M.A., Lelis I.C., DE Macedo M.F., Bezerra M.B. (2018). Hematological and Biochemical Profile of BALB/c Nude and C57BL/6 SCID Female Mice after Ovarian Xenograft. An. Acad. Bras. Ciênc..

[25] Botros M., Sikaris K.A. (2013). The De Ritis Ratio: The Test of Time. Clin. Biochem. Rev..

[26] Celise D.A., Kimotho J., Kimani J.W., Muriithi A.K., Odari E.O. (2023). Increase in the Immune Response in Balb/c Mice after the Co-Administration of a Vector-Based COVID-19 Vaccine with Cytosine Phosphoguanine Oligodeoxynucleotide. Vaccines.

[27] Washington I.M., Van Hoosier G. (2012). Clinical Biochemistry and Hematology. The Laboratory Rabbit, Guinea Pig, Hamster, and Other Rodents.

[28] Plumb D.C. (2008). Veterinary Drug Handbook.

[29] He S., Su G., Liu K., Zhang F., Jiang Y., Gao J., Xie H. (2017). Sex-specific reference intervals of hematologic and biochemical analytes in *Sprague*-*Dawley* rats using the nonparametric rank percentile method. PLoS ONE.

[30] Agbafor K.N., Nwaka A.C., Dasofunjo K., Asuk A.A., Ugwu M.M. (2017). Serum Activity of Alanine Aminotransferase and Aspartate Aminotransferase in Albino Rats Administered Aqueous Extract of Fresh Leaves *Pterocarpus santalinoids*. IDOSR J. Sci. Res..

[31] Ahmed B.M., Ali M.E., Masud M.M., Azad M.R., Naznin M. (2024). After-Meal Blood Glucose Level Prediction for Type-2 Diabetic Patients. Heliyon.

[32] Viriyavejakul P., Khachonsaksumet V., Punsawad C. (2014). Liver Changes in Severe *Plasmodium falciparum* Malaria: Histopathology, Apoptosis and Nuclear Factor Kappa B Expression. Malar. J..

[33] Arika W.M., Nyamai D.W., Musila M.N., Ngugi M.P., Njagi E.N.M. (2016). Hematological Markers of *In Vivo* Toxicity. J. Hematol. Thromboembolic Dis..

[34] Rosenblatt A.E., Stein S.L. (2015). Cutaneous Reactions to Vaccinations. Clin. Dermatol..

[35] Burlando M., Russo R., Cozzani E., Parodi A. (2021). COVID-19 “Second Wave” and Vaccines: The Dermatologists’ Perspective. Int. J. Dermatol..

[36] Murphy D.M., Cox D.J., Connolly S.A., Breen E.P., Brugman A.A., Phelan J.J., Keane J., Basdeo S.A. (2023). Trained Immunity Is Induced in Humans after Immunization with an Adenoviral Vector COVID-19 Vaccine. J. Clin. Investig..

[37] Adil M.S., Somanath P.R., Turksen K. (2020). Vascular Permeability Assays In Vivo. Permeability Barrier; Methods in Molecular Biology.

[38] Arimbi H., Plumeriastuti H., Widiyatno T.V., Legowo D. (2021). Textbook of General Veterinary Pathology [In Indonesian].

